# Characterization of the Omnivorous *Lygus lineolaris* Diet in a Strawberry Field by Metataxonomy

**DOI:** 10.1002/ece3.72954

**Published:** 2026-01-19

**Authors:** Mireia Solà Cassi, François Dumont, Eric Lucas

**Affiliations:** ^1^ Laboratoire de Lutte Biologique, Département des Sciences Biologiques Université du Québec à Montréal (UQAM) Montréal Québec Canada; ^2^ Centre de recherche agroalimentaire de Mirabel Mirabel Québec Canada; ^3^ Institute of Agrifood Research & Technology Barcelona Spain

**Keywords:** diet, food web interactions, metabarcoding, omnivory, pest management, tarnished plant bug

## Abstract

*Lygus lineolaris* is a highly polyphagous pest that impacts key crops such as strawberries, making an understanding of its feeding behavior critical for developing effective management strategies. Using metataxonomy, this study examined the dietary breadth of *L. lineolaris* in a commercial strawberry field in Quebec, revealing an extensive and diverse omnivorous diet. The multiprimer approach, combined with validation samples, ensured high taxonomic resolution and accuracy. We expanded the documented list of *L. lineolaris* host taxa to 475, including 441 plants and 34 prey species, with 51 taxa unique to this research, comprising eight new plant hosts and five prey species. Molecular evidence confirmed active ingestion, underscoring its omnivorous behavior with a predominantly herbivorous tendency. Notably, 70% of individuals fed exclusively on plants, 20% exhibited omnivory, and only 4% were strictly zoophagous. To quantify the level of phytozoophagy in omnivorous species, we propose a novel coefficient of omnivory (CO), calculated as CO = *P*/(*P* + Z), where *P* and *Z* represent the number of individuals with molecular evidence of phytophagy and zoophagy, respectively. With a CO of 0.833 (95% CI: 0.77–0.90), *L. lineolaris* demonstrates a strong bias toward plant feeding. Diet composition varied seasonally and between sexes, with females showing increased zoophagy during reproductive periods. These findings highlight *L. lineolaris* 's dietary flexibility and resilience, providing critical insights into its feeding ecology and food web interactions to inform targeted integrated pest management strategies tailored to its omnivorous nature.

## Graphical abstract

This study employs metataxonomy to reveal the extensive omnivorous diet of *Lygus lineolaris* in a strawberry field, identifying 475 host taxa and confirming active ingestion across plant and prey sources. We introduce a novel coefficient of omnivory, demonstrating the species' strong herbivorous bias while highlighting its dietary flexibility and seasonal, sex‐based variations. These findings enhance understanding of *L. lineolaris* 's feeding ecology and inform targeted pest management strategies.

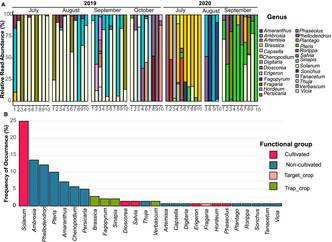

## 1 Introduction

*Lygus lineolaris* (Palisot de Beauvois) (Hemiptera: Miridae), commonly known as the tarnished plant bug (TPB), is the most prevalent *Lygus* species in Eastern North America and exemplifies the complexities of omnivorous behavior. With a distribution range from Mexico to Alaska (Al‐Ghamdi et al. 1995; Fleury et al. 2013; Holopainen and Hokkanen 2024), this polyphagous pest thrives on a wide variety of wild plants and crops, impacting over 130 economically significant species, including cotton, alfalfa, and strawberries, while also occasionally preying on other insects (Snodgrass et al. 1984; Young 1986; Esquivel and Mowery 2007; Wheeler 2011; George et al. 2021). Its geographical distribution is shaped by the availability of crops and weedy host plants, as well as agricultural practices (Hardman et al. 2004; Fleury et al. 2013). Furthermore, on a smaller scale, its feeding choices are influenced by resource availability, competition, predation risk, and sensory cues (Solà Cassi et al. 2025; Hetherington et al. 2025). These factors illustrate the complex interactions of omnivorous species within ecosystems and highlight the need for accurate characterization of their feeding behavior in situ. As a primary insect pest of strawberries in eastern and central North America, *L. lineolaris* poses significant risks to crop yields, necessitating effective monitoring and management strategies, especially during bloom and peak fruiting periods (Fleury et al. 2013; D'Ambrosio et al. 2019; George et al. 2021). *Lygus lineolaris* has gained significance in North America due to rapid population growth driven by high fecundity, short generation times, and a broad host range. Its adaptability to varying environmental conditions and resistance to insecticides have intensified its pest status (Blackmer et al. 2004; Wheeler 2011; Zhu et al. 2011; Showmaker et al. 2016; George et al. 2021). Additionally, the growing reliance on transgenic cotton which has reduced early‐season insecticide use has inadvertently boosted its proliferation (Armstrong and De Azevedo Camelo 2003; Layton 2000).

*Lygus lineolaris* is characterized as an early successional species that feeds on cultivated crops and surrounding weed species (Young 1986). It overwinters as an adult in ground litter and emerges in spring to feed on wild host plants near crops (Esquivel and Mowery 2007; Holopainen and Hokkanen 2024). As weeds senesce, *L. lineolaris* migrates into susceptible crops during their productive stages (Snodgrass et al. 1984; D'Ambrosio et al. 2019). All life stages of *L. lineolaris* damage reproductive structures, such as budding flowers and mature fruit, leading to issues like flower bud abscission, deformed fruits, necrosis, excessive branching, and stunted growth, which ultimately impact crop marketability (Strong and Sheldahl 1970; Tuelher et al. 2020).

Furthermore, based on the documented list of hosts, *Lygus lineolaris* has been ranked among the top nine most polyphagous arthropods (Thompson et al. 2023; Holopainen and Hokkanen 2024), with a recorded diet including 416 host plant and 20 prey taxa (Snodgrass et al. 1984; Young 1986; Wheeler 2011; Esquivel and Mowery 2007; Solà Cassi et al. 2024; Schwartz and Foottit 1992). Nevertheless, it is expected that the actual number of host plant species exceeds 700 (Parys and Snodgrass 2014). Its population dynamics are driven more by the quantity of available food than by its identity, with fitness gains influencing dietary choices (Andow 2023; Solà Cassi et al. 2024). Individual feeding specialization is further shaped by genetic, developmental, and sexual factors (Coll and Guershon 2002; Andow 2023). Moreover, physiological adaptations, such as specialized enzymes and mouthparts, allow *L. lineolaris* to thrive on an omnivorous diet throughout all developmental stages (Showmaker et al. 2016; Solà Cassi et al. 2024). Although most research has focused on plant hosts due to its pest status, TPB also exhibits facultative zoophagy, as reported for other mirids (Wheeler 2011; Solà Cassi et al. 2024). Available evidence indicates predation on insect eggs and soft‐bodied stages, and occasionally adults, though this aspect remains less documented. Access to prey has been shown to enhance fitness, supporting the ecological relevance of zoophagy alongside its predominantly phytophagous habits (Wheeler 2011; Solà Cassi et al. 2024).

Despite several studies on the hosts of *Lygus lineolaris* , a lack of consensus remains regarding the definition of a host (Snodgrass et al. 1984; Young 1986; Esquivel and Mowery 2007; Schwartz and Foottit 1992; Taksdal 1963). Generally, host plants are defined as species where *L. lineolaris* is present, with higher abundances of females or nymphs indicating their potential as reproductive hosts (Young 1986; Esquivel and Mowery 2007). However, there is still a lack of studies on *L. lineolaris* directly measuring feeding or oviposition hosts or studies with a thorough assessment of host plants within specific agricultural systems and regions, such as strawberry fields in Quebec. For instance, Solà Cassi et al. (2025) identified oviposition hosts by directly assessing *L. lineolaris* offspring in choice tests involving buckwheat [ *Fagopyrum esculentum* (Moench)], canola [ *Brassica napus* (L.)], and strawberries [ *Fragaria* × *ananassa* (Duchesne)]. They further demonstrated that *L. lineolaris* immatures fed on the flowering parts of these plants, either alone or in combination with aphids *Myzus persicae* (Hemiptera: Aphidae) (Sulzer) and spider mites *Tetranychus urticae* (Trombidiformes: Tetranychidae) (Koch), by providing these specific food sources during their development until adulthood to evaluate their effects on fitness (Solà Cassi et al. 2024). In parallel, Hagler et al. (2010) emphasized the value of molecular techniques to assess host plant associations under natural conditions, which are often difficult to capture through traditional observational methods. Their study on *Lygus hesperus* (Hemiptera: Miridae) (Knight), a closely related species to *L. lineolaris* , demonstrated how molecular approaches can reveal life stage‐specific feeding preferences in complex agroecosystems.

To characterize feeding interactions under field conditions, we used a metataxonomic metabarcoding approach, which allows the detection of ingested material and the reconstruction of trophic interactions from environmental DNA (eDNA) (Taberlet et al. 2007; Valentini et al. 2009; Pompanon et al. 2012; De Barba et al. 2014; Sow et al. 2020). This approach enables the parallel processing of many samples and provides reliable taxonomic assignments across a wide diversity of taxa (Wright 2024). However, metabarcoding remains constrained by biases such as incomplete reference databases, primer‐specific amplification differences, and the single‐locus nature of most public repositories (Valentini et al. 2009; Deagle et al. 2014; Alberdi et al. 2019; deWaard et al. 2019). To mitigate these limitations, we adopted a multiprimer, multilocus strategy supported by extensive validation samples and custom reference databases, an approach shown to reduce misidentification and increase taxonomic coverage (Piñol et al. 2015; Krehenwinkel et al. 2018; Elbrecht et al. 2019; Deagle et al. 2019; Jusino et al. 2019; Batuecas et al. 2024).

Using this framework, our first objective was to characterize the omnivorous dietary breadth of adult *L. lineolaris* in a commercial strawberry field in Quebec. Our second objective was to examine how diet varies by sex and across the season, allowing us to place the species within the continuum from phytophagy to zoophagy.

## 2 Materials and Methods

### 2.1 Experimental Design

#### 2.1.1 Field Samples

Adult tarnished plant bug (TPB) were collected from two organic experimental strawberry field in the Laurentides region of Quebec (Canada) during two consecutive summers (2019, 2020). In 2019, sampling was conducted at one location within the field (GPS: 45°37′55.9′′ N, 74°05′19.3′′ W), which consisted of 96 plots (2.5 m long and 1 m wide) with Albion variety strawberry plants, arranged in two lanes of 16 plants separated by 10 m. Wild plants, such as red‐rooted pigweed, *Amaranthus retroflexus* (L.) (Chenopodiaceae) and ragweed, *Ambrosia trifida* (L.) (Asteraceae) were preserved nearby, and TPB individuals were mostly collected from these wild plants using a mouth aspirator. In 2020, specimens were collected from a second sampling point using a mouth aspirator within the same field, located approximately 700 m from the 2019 site (GPS: 45°37′55.7′′ N, 74°04′50.2′′ W). This area was bordered by trap crops including mustard ( *Sinapis alba* ), canola, and buckwheat. These plant species, as well as *A. retroflexus* and *A. trifida* , have previously been reported as attractive to TPB. While the sampling plots differed between years, both fields are representative of realistic strawberry production systems in Quebec, either bordered by spontaneous vegetation (2019) or by trap crops (2020), which are increasingly used by growers to promote biodiversity and support natural enemies and pollinators. Given the assessed TPB dispersal capacity, flying more than 12 km in 12 h (Stewart and Gaylor 1994; Hagler et al. 2010), both sample fields were considered part of the same area. TPB adults were collected from late July to the onset of autumn (July–September 2019; July–October 2020), with the slight difference between years reflecting resource availability. Importantly, this timeframe corresponds to the full period during which *L. lineolaris* reaches peak abundance and causes economically significant damage in Quebec strawberry fields (Fleury et al. 2013; Handley and Pollard 1993). Each month, 10 individuals were collected and individually transferred to the laboratory (< 1 h) to be sexed by inspecting their genitalia under stereomicroscope and stored in a 1.5 mL microcentrifuge tube at −20°C until use (Table S1).

#### 2.1.2 Validation Samples

Validation samples included mock samples and feeding trial samples to assess primer efficacy and taxonomic resolution (Jusino et al. 2019). Mock samples consisted of known organisms and included four types: (i) Vegetal: cucumber [ *Cucumis sativus* (L.)] or buckwheat, (ii) Animal: three *M. persicae* or six syrphid legs [ *Eupeodes americanus* (Diptera: Syrphidae) (Wiedemann)], (iii) Mixed: a combination of all four types of organisms, and (iv) Negative control: a sample without DNA.

Feeding trial samples involved analyses of adult females of *L. lineolaris* and of *Nabis americoferus* (Carayon) (Hemiptera: Nabidae), a naturalized predator of *L. lineolaris* in strawberry fields of Quebec, through a choice test with known diets (Solà Cassi et al. 2025). The available food options included: (i) Animal: *T. urticae* or aphids *M. persicae* , (ii) Vegetal: strawberry, canola, and buckwheat, (iii) Mixed: a combination of all organisms, and (iv) Fasted: a negative control. The feeding trials were conducted in aerated arenas (60 × 40 × 30 cm) under controlled conditions (25°C, 18 h light, 70% relative humidity) for 3 days. Each arena contained three equidistant flowering‐stage plants, strawberry, canola, and buckwheat, infested with 50 *T. urticae* and *M. persicae* each. A total of 30 TPB, balanced for sex ratio, were included in each arena. Prior to the trials, each individual had access to water but was food‐deprived for 24 h to clear their guts (Solà Cassi et al. 2025). All validation samples came from our laboratory rearing's (see Table S2 for validation samples details).

### 2.2 Metataxonomy Pipeline

#### 2.2.1 DNA Extraction and Amplification

Specimens were washed to remove cuticle contaminants in two steps: first in 10 mL of DNA‐free water with 0.1% Tween 20 for 1 min, and then in DNA‐free water containing 0.5% sodium hypochlorite and 1% Tween 20 for an additional minute. Each insect was rinsed with DNA‐free water for 30 s and dried on filter paper.

DNA was extracted from the homogenized specimens using the Qiagen DNeasy Blood and Tissue Kit following the “animal tissue” protocol. Eluted DNA (30 μL AE buffer) was stored at −20°C. The concentration and quality of the extracted DNA were assessed using a Qubit 3.0 Fluorometer (Thermo Fisher Scientific, Waltham), and samples were normalized to a consistent concentration by adding sterile TE Buffer (10 mM Tris–HCl, 0.1 mM EDTA, pH 8.0) prior to further analysis.

A total of 93 samples were analyzed, comprising 70 TPB adults (43 females) collected from the field, 40 samples from 2019 (16 males and 24 females) and 30 (11 males and 19 females) from 2020. Additionally, 23 validation samples included 6 mock samples (1 replicate each) and 17 from feeding trials: 4 samples from *N. americoferus* with mixed diets and 13 from *L. lineolaris* , which consisted of 4 on mixed diets and 9 on single diets (e.g., animal, vegetal, or fasted), with 3 replicates for each food source (Tables S1 and S2).

#### 2.2.2 Primer's Selection

Primers were selected based on several criteria: they needed to amplify a wide range of plants and metazoans, successfully target highly degraded DNA, avoid overlapping in the same primer binding sites, and conform to the maximum read length of the MiSeq system (Illumina). Primer sets were chosen for their high representation in literature, success in taxon amplification, and suitability of amplicon size for sequencing, resulting in four primer sets targeting three regions (mitochondrial, chloroplast, and nuclear) (Table 1). This approach was intended to enhance detection sensitivity and resolution across trophic groups.

**TABLE 1:** Primer pairs used to detect *L. lineolaris* diet breath.

Target org.	Binding region	Primer name	Dir.	Primer sequence 5′–3′	References	Amplicon length
Animal	COI	mICOIintF	F	GGWACWGGWTGAACWGTWTAYCCYCC	Krehenwinkel et al. (2017)	365
		Fol‐degen‐rev	R	TANACYTCNGGRTGNCCRAARAAYCA		
		ZBJ‐ArtF1c	F	AGATATTGGAACWTTATATTTTATTTTTGG	Zeale et al. (2011)	157
		ZBJ‐ArtR2c	R	WACTAATCAATTWCCAAATCCTCC		
Vegetal	trnL	g	F	GGGCAATCCTGAGCCAA	Taberlet et al. (2007)	10–143
		h	R	CCATTGAGTCTCTGCACCTATC		
	ITS2	ITS‐u3	F	CAWCGATGAAGAACGYAGC	Cheng et al. (2016)	400

*Note:* Targeted organisms (plant or animal), targeted regions, primer's names, sequences (forward [F.] and reverse [R.]), references and amplicon length are indicated.

For metazoan identification, the cytochrome c oxidase subunit I (COI) region was targeted using ZBJ‐ArtF1c and ZBJ‐ArtR2 (Zeale et al. 2011) as well as mlCOIintF and Fol‐degen‐rev (Leray et al. 2013; Krehenwinkel et al. 2018). To identify plant species (Streptophyta), the universal P6 loop of the chloroplast trnL (UAA) intron was amplified using the g‐h primer set (Taberlet et al. 2007), while the nuclear ITS2 region was targeted with ITS‐u3 and ITS‐u4 (Chen et al. 2016) (Table 1). Performance differences among primers were evaluated (Tables S3–S5).

#### 2.2.3 PCR Amplification, Library Construction and Sequencing

Amplicon libraries were created using a dual, independent PCR approach, where each DNA sample underwent two separate reactions targeting different taxonomic groups: one for metazoans and one for plants (Streptophyta). Each reaction used two primer pairs: the metazoan PCR targeted the COI region, while the plant PCR targeted the ITS2 and trnL regions (Table 1). The PCR protocols were selected based on established literature and optimized in our laboratory to ensure robustness and reproducibility, addressing known limitations such as primer bias and incomplete reference databases.

All PCR reactions were performed in triplicate in a 25 μL volume containing 1× Buffer HF, 0.2 mM dNTPs mix, 3% DMSO, 0.4 μM of each primer, 0.02 U/μL Phusion Hot Start II DNA polymerase, and 1 μL of DNA template. Negative controls were included to monitor contamination.

PCR conditions for metazoan DNA involved 33 cycles: denaturation at 98°C for 30 s, annealing at 46°C for 30 s, extension at 72°C for 30 s, and a final extension at 72°C for 10 min. PCR conditions for Streptophyta involved 40 cycles with denaturation at 98°C for 30 s, annealing at 55°C for 30 s, extension at 72°C for 30 s, and a final extension at 72°C for 10 min. Amplifications were conducted using an Eppendorf Mastercycler Nexus GSX1.

After PCR, libraries were formed by combining the three PCR replicates for each sample into one sequencing library per target group, each containing 84 samples (Table S2), and processed in a single high‐throughput sequencing run. Dual indexes and sequencing adapters (Nextera; Illumina) were added to the PCR products to assign unique sample identifiers for bioinformatics analysis.

Quality control steps included visualizing PCR products on an agarose gel, normalization using CharmBiotech's Just‐a‐plate 96 PCR purification and normalization protocol, and purification with the NucleoMag kit (Macherey‐Nagel). Libraries were quantified using the Qubit dsDNA HS Assay Kit (Invitrogen) and NEBNext Library Quant Kit (New England BioLabs), with average fragment size assessed using the Agilent TapeStation 4200. Fragment sizes for each primer set are reported in Table S4. To address sequencing depth, a Phix control library (Illumina) was incorporated. Differences in the number of sequencing libraries between years reflect the sampling effort, as 2019 sampling extended into October while 2020 sampling ended in September due to resource constraints, resulting in fewer individuals and fewer libraries.

Sequencing was performed on an Illumina MiSeq at CERMO‐FC Genomic Platform, Université du Québec à Montréal, using a MiSeq reagent kit v3 with paired‐end 2 × 300 bp reads (600 cycles).

#### 2.2.4 Custom Database

A custom database of Amplicon Sequence Variants (ASVs) was constructed of each target region: trnL and ITS2 for Streptophyta, and COI for Metazoans. Sequences were retrieved from the NCBI server using the NCBI Mass Sequence Downloader tool in February 2023. The database construction involved removing duplicates, addressing missing data, and filtering sequences by specific lengths. No similarity thresholds were applied during database construction (Table S3). Taxonomic rankings were added using the taxonomizr (Sherrill‐Mix 2025). Retained sequence lengths were based on barcode literature for the selected regions (Taberlet et al. 2007; Yao et al. 2010; Ruegger et al. 2011; Elbrecht et al. 2019; Ballin et al. 2019; Santos et al. 2020; Timpano et al. 2020; Foster et al. 2021; Barnes et al. 2022) (Table S3). Taxonomic classifications were assigned by training the “LearnTaxa” function of the Decipher package (Wright 2016) with 10 sequences per group, repeated five times. A total of 98,220, 77,503, and 373,534 groups were retained for the trnL, ITS2, and COI regions, respectively (Table S3).

#### 2.2.5 Bioinformatic Analyses

Bioinformatic processing of sequencing reads involved separate analyses for each primer set. After demultiplexing by the sequencing facility, primers were identified and separated using the BBTools suite (BBDuk, bbmap 38.86) (Bushnell 2022) and removed with Cutadapt 2.10 (Martin 2011) using default mismatch parameters and anchored adapter trimming. PCR negative controls were included for each primer set and sequenced together with samples to enable contamination detection using the decontam package. Quality control included inspecting read profiles and truncating reads based on quality scores (truncLen = 150–250 depending on primer set). Reads were filtered by maximum expected errors (maxEE = 1.1 for trnL g/h and ZBJ‐ArtF1C/ZBJ‐ArtR2C; 2.2 for ITS‐u3/ITS‐u4 and mlCOIintF/Fol‐degen‐rev), applied to both forward and reverse reads. Reads shorter than 10 nucleotides for *trnL* and 50 nucleotides for ITS2 and COI were excluded (Yang et al. 2021). Maximum expected error thresholds were adjusted by primer set to account for differences in amplicon length and quality profiles (Callahan et al. 2016).

Denoising, merging, and chimera removal were performed using DADA2 (v1.22.0) (Callahan et al. 2016). Merging required a minimum overlap of 5 bp for *trnL* and 10 bp for other markers, reflecting the short length of *trnL* amplicons and standard Illumina paired‐end practice. Strict mismatch criteria were applied: 0 for most primer sets and 1 for COI to reduce misassemblies.

Taxonomic assignment used IDTAXA with a confidence threshold of 60 (Murali et al. 2018). Post‐processing removed singletons and contaminants using decontam package (v1.14.0) 1.14.0 package with thresholds of 0.3 for trnL and 0.5 for ITS2 and COI (Davis et al. 2018; Drake et al. 2022). Bias mitigation included removing conspecific reads for *L. lineolaris* and *Nabis americoferus* and excluding taxa with lower read counts than those detected in PCR negative controls (Piñol et al. 2014; Drake et al. 2022).

### 2.3 Statistical Analysis

Only ingested taxa identified below family level of field‐collected individuals were included in the statistical analyses. Relative read abundance (RRA%) was calculated and visualized, filtering out identifications < 1% RRA (Deagle et al. 2019). The frequency of occurrence (FOO%), which measures how often a particular taxon is detected across samples, for each taxon was assessed for both the last taxonomical level identified and for the taxa identified at the Genus level. For both plant and animal identification, taxa were then consolidated using a Python script to ensure unique taxa per sample and address duplicates by merging less resolved taxa with higher resolved ones from complementary markers (da Silva et al. 2019). Streptophyta were categorized according to the type of plant (e.g., herbaceous, tree, fern), and their functional group (non‐cultivated, cultivated, trap crop, target crop). This classification was performed with GBIF (https://www.gbif.org/), IRIIS (https://www.iriisphytoprotection.qc.ca/), and on‐site visits. Algae, mosses, and unlikely plants were excluded or reclassified at lower taxonomic levels (more details in Supporting Information). Metazoans were categorized as natural enemies (predators and parasitoids), herbivores, pests, omnivores, and others (e.g., blood feeders, scavengers).

The number of occurrences, as well as richness and Shannon diversity, were calculated separately for individual specimens, for diet type (vegetarian, animal, or omnivorous) and for each period (month and year). Differences in sex ratio and diet type were analyzed using the chi‐squared test, while Fisher's exact test was employed when any expected cell count was less than 5. Subsequently, pairwise comparisons with an alpha level of < 0.05 were conducted to evaluate differences among the levels.

Several Generalized Linear Mixed Models (GLMMs) were conducted, all incorporating year as a random effect. First, the proportion of zoophagous individuals relative to phytophagous ones was analyzed using a GLMM with a binomial distribution, with fixed factors including sex and month. The same fixed factors were applied to study total occurrences using a GLMM with a Poisson distribution. This model was also employed to analyze the number of phytophagous occurrences, with month as a factor. For zoophagous occurrences, a zero‐inflated model with a Poisson distribution and a random intercept for year was utilized.

Data was transformed to analyze the effects on taxa richness and diversity. Abundances were standardized using the Hellinger method to address double‐zero issues, followed by Tukey's power transformation to ensure normality and homoscedasticity. Standardization allowed for a mean of 0 and a standard deviation of 1. A linear mixed model was then used to evaluate the effects of month on taxa richness and diversity of items ingested, with year included as a random effect.

Selected models were those with the smallest AIC value. To assess overdispersion, deviance for models with a Poisson distribution was evaluated. Models were verified and adjusted accordingly through examination of diagnostic plots, including residual plots and histograms of residuals. Subsequently, post hoc tests with Bonferroni correction were performed. Post hoc test statistical results are not included to enhance readability.

To assess differences in community composition of both phytophagous and zoophagous individuals across months and years, a presence‐absence matrix of taxa was created for each sample and standardized using the Hellinger method. Bray–Curtis similarity was then calculated between samples, and differences in community composition were evaluated using permanova with 999 permutations.

Analyses were conducted in R (version 4.5.1) (R Core Team 2021) using the following packages: *tidyverse* (2.0.0), including *dplyr* (1.1.4), *tidyr* (1.3.1), *readr* (2.1.5), *stringr* (1.5.2), and *forcats* (1.0.0); *ggplot2* (4.0.0) for visualization; *caret* (7.0‐1) and *e1071* (1.7‐16) for machine learning; *lme4* (1.1‐37), *glmmTMB* (1.1.13), and *brms* (2.23.0) for mixed‐effects and Bayesian modeling; *emmeans* (2.0.0) and *multcomp* (1.4‐28) for post hoc tests; *vegan* (2.7‐1) for ecological analyses; and packages for metabarcoding data processing and analysis, including *dada2* (1.38.0), *DECIPHER* (3.6.0), *ShortRead* (1.68.0), *Biostrings* (2.78.0), *phyloseq* (1.54.0), and *decontam* (1.30.0). Additional packages used for visualization and reporting included *cowplot* (1.2.0), *ggpubr* (0.6.1), *kableExtra* (1.4.0), and *RColorBrewer* (1.1‐3). For all statistical tests, the significance level was set at *α* = 0.05.

### 2.4 Coefficient of Omnivory

To classify omnivorous species along the phytozoophagous dietary continuum, we used the coefficient of omnivory (CO), a novel quantitative metric defined as: CO = *P*/(*P* + Z), where (*P*) is the number of individuals with molecular evidence of phytophagy, (*Z*) is the number with molecular evidence of zoophagy. Individuals with both plant and animal detections were assigned *P* = 0.5 and *Z* = 0.5. Thus, CO ranges from 1 (strictly phytophagous) to 0 (strictly zoophagous). This approach provides a continuous measure of feeding behavior rather than a binary classification.

For robust and comparable CO estimates, we recommend: (i) Molecular data on feeding behavior, (ii) sampling across the full season with ≥ 3 dates and similar numbers of individuals per date, (iii) sex‐specific representation, and (iv) a minimum of 50 individuals. For species‐level estimates at broader scales, replication across ≥ 3 distinct regions is advised. To account for variability, CO should be reported with bootstrap confidence intervals and metadata (sex, stage, site, season).

We estimated CO for *Lygus lineolaris* in the Laurentides region using metabarcoding data from 70 individuals collected across the full season over 2 years (10 individuals per month). The experimental design aimed to ensure temporal coverage and sex representation, although sex was determined post hoc and was unevenly distributed across months. Uncertainty in CO was quantified using bootstrap resampling (*B* = 2000 iterations). Individuals were resampled with replacement from observed feeding states (phytophagous = 1, zoophagous = 0, omnivorous = 0.5). We computed: (i) Global CO using stratified resampling by month × year, preserving stratum sizes, (ii) sex‐specific CO using stratification by month × sex. Because one stratum (July–male) had only four individuals, we repeated the analysis with stratification by month only as a sensitivity check. Strata with *n* ≥ 3 were considered acceptable (*n* ≥ 5 ideal).

Sex differences were evaluated using a month‐constrained permutation test (*B* = 10,000), which permutes sex labels within months to control for temporal structure.

## 3 Results

### 3.1 Primer Resolution

#### 3.1.1 Streptophyta

**FIGURE 1:**
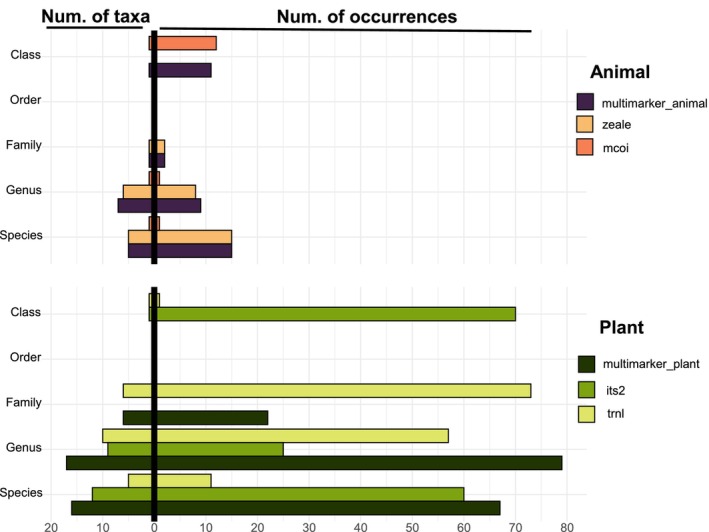
Taxonomic resolution of primers based on the number of taxa identified at each taxonomic level ingested by *L. lineolaris* collected in the field (left), and the number of occurrences observed at each taxonomic level (right) for animal (top) and plant (bottom) taxa, categorized by the marker used. For animals, ZBJ‐ArtF1c/ZBJ‐ArtF2c and mICOIintF/Fol‐degen‐rev markers both targeting the mitochondrial COI region were used as well as their combination in a multimarker approach (multimarker_animal). For plants, the marker g/h targeting the chloroplast trnL region and the marker ITSu3/ITSu4 targeting the ITS2 nuclear region were used in addition to the multimarker approach.

The g/h primer retained twice as many reads compared to the ITS‐u3/ITS‐u4 primer after quality filtering, although g/h lost 9% more reads after filtering by controls (Table S4). While the ITS‐u3/ITS‐u4 primer yielded 190 more occurrences than g/h primer, 55.07% of these were classified only at or above the Class level, compared to 17.25% for g/h. In field samples, both primers identified a similar number of taxa beyond Class (*n* = 21), but their resolution varied. The ITS‐u3/ITS‐u4 primer identified 52.17% of occurrences at the species level, more than double that of g/h, which provided greater identification at the genus and family levels. The use of plant multimarkers doubled the number of identified taxa to 40, with approximately 40% classified at the species level and 40% at the genus level (Figure 1; Table S4).

#### 3.1.2 Metazoan

Both metazoan primer sets exhibited similar read loss after quality filtering, ranging from 10% to 13%. However, ZBJ‐ArtF1c/ZBJ‐ArtF2c produced nearly eight times more reads per sample than mICOIintF/Fol‐degen‐rev. After filtering out self‐host DNA, both primers lost over 80% of their reads. In field samples, ZBJ‐ArtF1c/ZBJ‐ArtF2c identified double the occurrences compared to mICOIintF/Fol‐degen‐rev, with 25 occurrences across 12 distinct taxonomic groups, while mICOIintF/Fol‐degen‐rev detected only two different taxa beyond Class level. Animal multimarkers resulted in 27 occurrences identified beyond class level spanning 13 unique Arthropoda taxa beyond Class level, with 35.71% identified at the species level, 50.00% at the genus level, and 7.14% at both the family and class levels (Figure 1; Table S4).

Across both animal and plant datasets, order‐level assignments were markedly fewer than class or genus assignments (Figure 1). This reflects that the short and degraded dietary fragments often provide enough information for broad (class) or fine (genus/species) matches, while intermediate ranks receive fewer confident assignments from the classifier.

Agreement between primers (ITS2 vs. trnL for plants; zeale vs. mcoi for animals) was quantified per item at five taxonomic levels and summarized in Table S5 together with all conflicting assignments (Table S6).

### 3.2 Validation Samples

Validation samples were conducted to evaluate the accuracy of the metataxonomy. This process confirmed the reliability of the identified taxa from *L. lineolaris* collected in the field and revealed the under detection of some species, contamination of others, and instances of secondary predation. More specifically, *Fagopyrum* sp. was detected in the mock samples but *Cucumis* sp. was not. In feeding trials, *Fagopyrum* sp., *Brassica* sp. (L.), and *Fragaria* sp. were ingested by *Nabis* and *Lygus*, with *Fragaria* sp. being the least frequently detected at 33% compared to trap crops, which were detected in over 60% of samples. Some cross‐contamination was also observed (see Supporting Information for detailed information) (Table S2; Figure S1).

All metazoans in the mock samples were successfully identified when examined individually; however, *Myzus* sp. was absent in the mixed samples. Additionally, three common biological contaminants from our greenhouses were identified (see Supporting Information for detailed information) (Table S2; Figure S1).

### 3.3 Field Samples

#### 3.3.1 Type of Diet

Most adults were phytophagous (70%), with fewer omnivorous (20%) and zoophagous (4%) individuals ($\chi_{2}^{2}=50.174$, *p* < 0.001). Among those consuming animal matter, most were omnivorous. The sex ratio was balanced in both years (0.40 in 2019; 0.37 in 2020; $\chi_{1}^{2}=3.657$, *p* = 0.056), though July was female‐biased (80% females). Diet type varied significantly by sex and month (LRT_1_ = 5.933, *p* < 0.05; LRT_3_ = 9.337, *p* < 0.05), with females more likely to prey (73% of zoophagous individuals). Zoophagy was four times more frequent in September than in July, when predation was rare and most individuals were phytophagous (Table 2).

**TABLE 2:** Phytophagous and zoophagous occurrences (Freq.), alpha diversity metrics (Richness, Shannon Diversity), of ingested hosts by *L. lineolaris* across seasons (year and month).

Period		Phytophagy						Zoophagy					
Year	Month	Freq.	*R*	*H*	% Fed	% Strict diet	% F eat	Freq.	*R*	*H*	% Fed	% Strict diet	% F eat
2019	7	22	13	2.51	100	90	80	1	1	0	10	0	100
	8	23	5	1.54	100	80	50	2	2	0.69	20	0	100
	9	36	14	2.53	100	50	60	9	8	2.07	60	0	83
	10	22	11	2.33	90	78	55	2	2	0.69	20	0	50
2020	7	29	14	2.59	100	90	80	1	1	0	10	0	100
	8	9	5	1.58	50	75	80	8	6	1.77	40	75	100
	9	27	10	2.14	100	70	50	3	1	0	30	0	33

*Note:* Additionally, the percentage of individuals that strictly consumed plants (phytophagy) or animals (zoophagy) (% strict diet) and the proportion of females among the individuals that engaged in that specific dietary intake (% F eat) are presented.

Taxa occurrences varied significantly by month but not by sex (LRT_3_ = 10.527, *p* < 0.05; LRT_1_ = 0.639, *p* = 0.424). Predation peaked in August 2020, when all strictly zoophagous individuals were collected, all females. This month recorded the lowest phytophagy rates, with no omnivores and the highest incidence of undetected taxa (*n* = 3). September had the highest cumulative occurrences (75) and Shannon diversity (LRT₃ = 9.950, *p* < 0.05), particularly in 2019, along with the greatest proportion of omnivorous individuals (50%). However, month and sex did not significantly affect per‐individual richness ($\chi_{4}^{2}=7.492$, *p* = 0.112) (Table 2).

#### 3.3.2 Phytophagy

**FIGURE 2:**
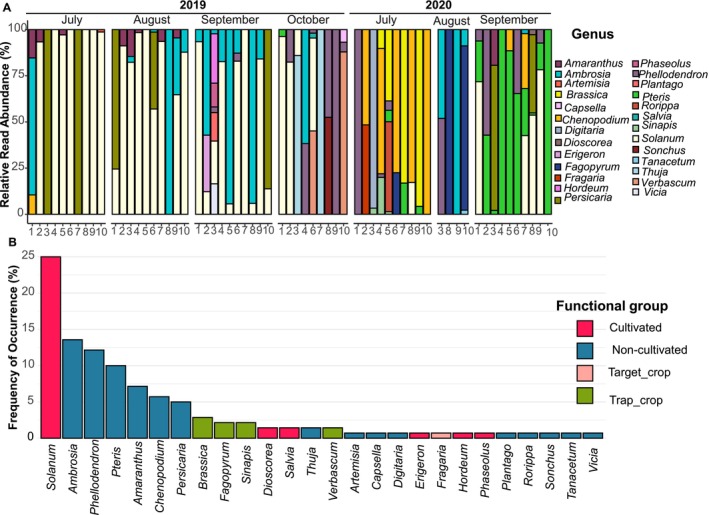
Genus‐level (A) relative read abundance (%RRA) and (B) frequency of occurrence (%FOO) of Streptophyta taxa detected by a multimarker metataxonomic approach in field‐collected *L. lineolaris* during two production seasons. (A) Sample labels indicate the individual number (1–10) within each monthly sampling. (B) Functional groups are displayed in different colors. The “Target crop” category, within the cultivated crop functional group, refers exclusively to strawberry.

Plant DNA was detected in 93% of individuals, spanning 37 taxa (29 below genus level), mostly herbaceous (87%). Non‐cultivated plants dominated (60%), followed by cultivated species (27%, including strawberry) and four trap crops (Table 3; Figure 2). Individuals ingested 1–9 plant taxa, with significant seasonal variation (*F*_month*year_ = 4.25, *p* < 0.001). In 2019, *Solanum* spp. (L.) dominated (75% of individuals), followed by *Ambrosia* spp. (L.) (40%, mainly September) and *Persicaria* spp. (Mill.) (16%, from July to September). *Amaranthus* spp. (L.) was frequent early in the season (20%), while *Phellodendron amurense* (Rupr.) appeared late (17.5%) (Table 3; Figure 2). In 2020, diets also varied across months. In July, *Chenopodium* spp. (L.) (50%) and *Brassica* spp. (L.) (40%) dominated, with strawberry detected only once, mixed with *Chenopodium* spp. In September, *Pteris* spp. (L.) occurred in 90% of individuals, mostly combined with *P. amurense* (50%) and other taxa such as *Solanum* spp., *Persicaria* spp., and *Amaranthus spp*. (44%, 22%, and 11%, respectively) (Table 3; Figure 2).

**TABLE 3:** List of plant taxa identified from *L. lineolaris* collected in the strawberry field using *metataxonomy*.

Class	Order	Family	Species	Sum reads	Freq.	Type of plant	Functional group
Magnoliopsida	Asterales	Asteraceae		16,787	4	—	—
			*Ambrosia* spp. (L.)	225	4	Herbaceous	Non‐cultivated
			*A. artemisiifolia* (L.)	4822	19		
			*Artemisia* sp. (L.)	35	1		
			*Erigeron canadensis* (L.)	190	1		Cultivated
			*Sonchus* spp. (L.)	126	1		Non‐cultivated
			***Tanacetum* spp. (L.)**	29	1		
	Brassicales	Brassicaceae		16,773	5		—
			*Brassica* spp. (L.)	3157	4		Trap crop
			*Capsella bursa‐pastoris* (L.)	166	1		Non‐cultivated
			*Rorippa* spp. (Scop.)	1155	1		Non‐cultivated
			***Sinapis alba* (L.)**	1242	3		Trap crop
	Caryophyllales	Amaranthaceae	*Amaranthus* spp. (L.)	2106	10		Non‐cultivated
		Chenopodiaceae	*Chenopodium* spp. (L.)	17,990	8		
		Polygonaceae		9110	1		—
			*Fagopyrum* spp. (Mill.)	4380	3		Trap crop
			*Persicaria* spp. (Mill.)	19,615	5		Non‐cultivated
			*P. lapathifolia* (L.)	105	2		
			***P. longiseta* (Bruijn)**	107	1		
	Dioscoreales	Dioscoreaceae	***Dioscorea alata* (L.)**	94	1		Cultivated
			***D. japonica* (Thunb.)**	39	1		
	Fabales	Fabaceae		3576	2		—
			*Phaseolus vulgaris* (L.)	730	1		Cultivated
			** *Vicia faba* (L.)**	935	1		
	Lamiales	Lamiaceae	***Salvia nemorosa* (L.)**	120	2		
		Plantaginaceae	*Plantago* spp. (L.)	876	1		Non‐cultivated
		Scrophulariaceae	*Verbascum* spp. (L.)	3632	2		Trap crop
	Poales	Poaceae		16,168	8		—
			***Digitaria* spp. (Haller)**	1315	1		Non‐cultivated
			***Hordeum murinum* (L.)**	1515	1		
	Rosales	Rosaceae		10,172	2		—
			*Fragaria* spp. (L.)	759	1		Target crop
	Sapindales	Rutaceae	** *Phellodendron amurense* (Rupr.)**	1643	17	Tree	Non‐cultivated
	Solanales	Solanaceae	*Solanum* spp. (L.)	51,760	35	Hebaceous	Cultivated
Pinopsida	Cupressales	Cupressaceae	**Thuja spp**. **(L.)**	11,802	2	Tree	Non‐cultivated
Polypodiopsida	Polypodiales	Pteridaceae	**Pteris spp**. **(L.)**	196	2	Fern	Non‐cultivated
			***P. cretica* (L.)**	2394	11		
			***P. vittata* (L.)**	147	1		

*Note:* The sum of reads and the number of total occurrences (Freq.) are described. Additionally, for identifications below genus level, the type of plant (e.g., herbaceous, tree, fern) and their functional group (non‐cultivated, cultivated, trap crop, target crop) are described. The first mention of each plant feeding host taxon is in bold.

Only 24% of phytophagous adults fed on a single plant genus, half of them in July (Figure 2). *Solanum* spp. was most frequent among these (10%), followed by *Persicaria* spp., *Ambrosia* spp., and *Pteris* spp. (3% each). Other single‐plant taxa included *Digitaria* spp. (*Haller*), *Chenopodium* spp., and *Fagopyrum* spp. (1.6% each). These taxa showed the highest read counts, especially *Solanum* spp. Co‐occurrence patterns were common: *Solanum* spp. often paired with *Ambrosia* spp. or *Amaranthus* spp., while *Ambrosia* spp. frequently mixed with *P. amurense*. Most genera (61%) had a frequency of occurrence (FOO) below 2% (Figure 2).

#### 3.3.3 Zoophagy

**FIGURE 3:**
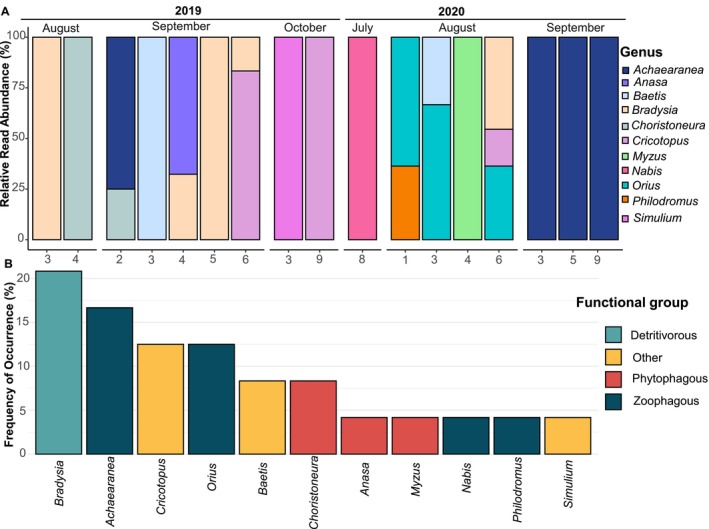
Genus‐level (A) relative read abundance (%RRA) and (B) frequency of occurrence (%FOO) of Metazoa taxa detected by a multimarker metataxonomic approach in field‐collected *L. lineolaris* during two production seasons. (A) Sample labels indicate the individual number (1–10) within each monthly sampling. (B) Functional groups are displayed in different colors.

Animal DNA was detected in 27% of individuals. Among consumed metazoan taxa, 42% were zoophagous species, 27% phytophagous, 27% detritivores, and 18% belonged to other feeding types, including hematophagous (Table 4). Most predatory events (65%) involved a single taxon, while 35% included two taxa, and only one individual contained DNA from three animal taxa (Figure 3). *Lygus lineolaris* (TPB) exhibited a diverse predatory pattern, consuming 13 different animal taxa. Composition varied by month and year (*F*_month*year_ = 1.71, df = 2, *p* < 0.01). Most prey belonged to Class Insecta, with two arachnid taxa from distinct families (Table 4). Among insects, four taxa were Diptera (three families), six were Hemiptera, and one Ephemeroptera species, *Baetis fuscatus* (L.), accounted for 8% of occurrences (Figure 3). The majority of reads were attributed to Miridae (Hahn), the family of TPB (Table 4). The arachnid *Achaearanea tabulate* (Levi) had the highest read count and was the second most frequent taxon (Figure 3). At the genus level, *Bradysia* spp. (Winnertz) was most frequent, while the predator *Orius insidiosus* (Say) appeared in August 2020 (*n* = 3), alongside two or three other taxa (Figure 3). The hematophagous *Simulium* spp. (Latreille), the predator *Nabis* spp. (Costa), and the phytophagous pest *Myzus* spp. (Passerini) were each detected only once and singly, in October 2019, July 2020, and August 2020, respectively (Figure 3).

**TABLE 4:** List of metazoa taxa identified from *L. lineolaris* collected in the strawberry field using *metataxonomy*.

Class	Order	Family	Species	Sum of reads	Freq.	Functional group
Arachnida	Araneae	Philodromidae	***Philodromus* spp. (Walckenaer)**	4	1	Zoophagous
		Theridiidae	***Achaearanea tabulata* (Levi)**	31	4	
Insecta	Diptera	Chironomidae	***Cricotopus annulator* (Goetghebuer)**	13	3	Detritivorous
		Sciaridae	***Bradysia* spp. (Winnertz)**	14	2	
			***Bradysia ocellaris* (Comstock)**	7	3	
		Simuliidae	***Simulium* spp. (Latreille)**	4	1	Other
	Ephemeroptera	Baetidae	***Baetis fuscatus* (L.)**	9	2	
	Hemiptera	Anthocoridae	***Orius insidiosus* (Say)**	15	3	Zoophagous
		Aphididae	***Myzus* spp. (Passerini)**	1	1	Phytophagous
		Coreidae	***Anasa* spp. (Amyot & Serville)**	23	1	
		Miridae		5910	2	—
		Nabidae	***Nabis* spp. (Latreille)**	1	1	Zoophagous
	Lepidoptera	Tortricidae	***Choristoneura* spp. (Lederer)**	3	2	Phytophagous

*Note:* The sum of reads, the number of total occurrences (Freq.), and the functional group is described. The first mention of each prey taxon is in bold.

#### 3.3.4 Omnivory

**FIGURE 4:**
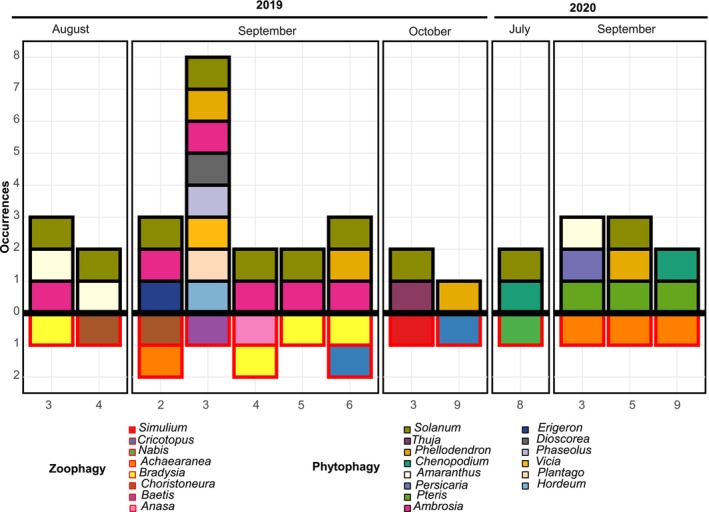
Omnivory‐related occurrences detected per *L. lineolaris* individual, categorized as zoophagy (Metazoa; values below zero, red outlines) or phytophagy (Streptophyta; values above zero, black outlines). Bars represent genus‐level detections in each individual sample, with colors indicating the contributing genera. Samples are grouped by month and year of collection. Sample labels indicate the individual number (1–10) within each monthly sampling.

Omnivory was detected in 15 individuals, which showed richer diets (4.0 ± 0.55 taxa per individual) than strictly phytophagous (2.0 ± 0.15) or zoophagous (2.0 ± 0.41) individuals. Most omnivorous events occurred in 2019 (73%), with a marked peak in September (60%). Co‐occurrence patterns were driven by a few recurrent animal–plant combinations. Among animal taxa, *Bradysia* spp. and *A. tabulata* (*n* = 4 each) and *Choristoneura* spp. (*n* = 3) were the most frequent. These co‐occurred primarily with *Solanum* spp. (*n* = 10), *Ambrosia* spp. (*n* = 6), *Amaranthus* spp. (*n* = 3), and *Pteris* spp. (*n* = 3) (Figure 4).

*Bradysia* spp. always appeared together with *Ambrosia* spp. and *Solanum* spp. (100% co‐occurrence). *Choristoneura* spp. also co‐occurred consistently with *Solanum* spp. (100%) and occasionally with other plant taxa. *A. tabulata* was paired with *Pteris* spp. in 75% of its occurrences. *Cricotopus* spp. was consistently associated with *P. amurense* (Figure 4).

### 3.4 Coefficient of Omnivory (CO)

The global coefficient of omnivory (CO) for *Lygus lineolaris* in the Laurentides region was 0.833 (95% bootstrap CI: 0.768–0.891), indicating a predominantly phytophagous feeding pattern. Sex‐specific estimates were 0.791 (0.709–0.872) for females and 0.904 (0.808–0.981) for males. The difference between sexes was −0.113 (female minus male), with a 95% bootstrap CI of −0.229 to 0.018. A month‐constrained permutation test yielded *p* = 0.159, indicating no statistically significant difference between sexes. Sensitivity analyses using month‐only stratification produced nearly identical CIs, supporting robustness against sex × month imbalance.

## 4 Discussion

Using metataxonomy, we characterized the diet of the highly polyphagous pest *Lygus lineolaris* in a commercial strawberry field in Quebec, expanding its host list and quantifying effects of sex and sampling period on adult feeding preferences.

We documented an extensive dietary breadth of 475 host taxa, including 441 documented plants and 34 prey. In Quebec strawberry fields, 51 unique taxa were identified, comprising 38 *Streptophyta* (resolved beyond family level) and 13 metazoans, including five species‐level identifications. These data tentatively extend the *L. lineolaris* host list with 13 plant taxa and 12 prey taxa not previously reported.

Pre‐extraction washing reduced detection of surface contaminants and foraged items (Batuecas et al. 2024; Huszarik et al. 2023), though incidental ingestion and non‐feeding contact cannot be fully excluded; associations should be interpreted cautiously. Additionally, in predatory individuals, some plant detections may reflect secondary ingestion via prey gut contents (de Bruyn et al. 2021). This approach, consistent with prior work, focuses on ingested taxa and potential feeding interactions. Within this context, *L. lineolaris* included 11 previously reported hosts, six newly documented prey species, and seven additional animal taxa assigned at higher ranks. Minor year‐to‐year differences (2019 vs. 2020) may reflect variation in surrounding vegetation rather than biological shifts because fields were nearby but not identical.

*L. lineolaris* feeds mainly on herbaceous plants but also on trees and ferns; 60% of *Streptophyta* detections were non‐cultivated, indicating a complex diet spanning hosts with diverse nutrients and defenses (Young 1986; Esquivel and Mowery 2007; D'Ambrosio et al. 2019). It also preys on pests such as *Frankliniella occidentalis* (Thysanoptera: Thripidae) (Pergande), potentially benefiting crops, while consuming natural enemies like *Orius insidiosus* (Hemiptera: Anthocoridae) (Say) and *Nabis* spp. Although predation is a small fraction of the diet and *L. lineolaris* often functions as prey, especially under shared‐resource, intraguild contexts, this omnivory underscores complex ecological interactions.

Significantly more adults were strictly phytophagous than omnivorous or zoophagous, positioning *L. lineolaris* toward the phytophagous end of the phytozoophagous continuum. The bootstrap‐based coefficient of omnivory (CO) for the Laurentides population was 0.833 (95% CI: 0.768–0.891), indicating a strong overall bias toward plant feeding despite notable animal consumption. To capture species‐level patterns globally, we recommend applying the CO framework across at least two additional regions.

Seasonal dynamics showed predominantly phytophagous diets in July, whereas September had the highest incidence of animal consumption, coincident with peak plant ingestion and many individuals ingesting both plant and animal matter. Most prey consumption occurred within omnivory, not strict zoophagy.

Sex‐specific patterns indicated greater zoophagy in females (CO = 0.791, 95% CI: 0.709–0.872) than males (CO = 0.904, 95% CI: 0.808–0.981), particularly from August to September. This aligns with Canadian phenology, first‐generation females carrying chorionated eggs and second‐generation females entering reproductive diapause (Gerber and Wise 1995) and likely reflects elevated energetic demands for reproduction and overwintering, consistent with Solà Cassi et al. (2024) showing fitness gains with animal prey. Late‐season resource dynamics may also contribute: strawberry remains available while senescing naturalized hosts reduce plant resources, potentially prompting greater zoophagy. Together, physiology and resource availability jointly shape *L. lineolaris* feeding.

A robust experimental design supported these findings: extensive mock and feeding‐trial validation samples and a multiprimer strategy drawn from the literature enhanced taxonomic resolution and reliability for field‐collected adults (Piñol et al. 2015; Krehenwinkel et al. 2017; Batuecas et al. 2022; Deagle et al. 2019). Design refinements minimized misclassification and improved detection, enabling insights into food web interactions critical for integrated pest management (Eubanks and Denno 2000; Hagler et al. 2010; Goeiz Pearson et al. 2011; Ugine et al. 2019). Our four‐primer, three‐region strategy (two plant, one animal) doubled *Streptophyta* identifications and stabilized animal detection when mICOIintF/Fol‐degen‐rev underperformed. The ZBJ‐ArtF1c/ZBJ‐ArtR2c pair produced nearly 8× more reads per sample than mICOIintF/Fol‐degen‐rev. Future work may benefit from alternatives such as ANML (LCO1490/CO1‐CFMRa), which yielded nearly 4× more arthropod taxa (Jusino et al. 2019), or mlCOIintF/HC02198 (Folmer et al. 1994), which amplified 20% more samples than ZBJ (Batuecas et al. 2022, 2024). For *Streptophyta*, the g–h primer targeting chloroplast trnL (Taberlet et al. 2007) outperformed ITS‐u3/ITS‐u4 (nuclear ITS; Chen et al. 2016), retaining 2× more reads post‐filtering, although ITS‐u3/ITS‐u4 achieved higher species‐level identifications, as seen elsewhere (De Barba et al. 2014; Cheng et al. 2016; Alberdi et al. 2019).

Despite filtering to reduce misidentifications from scavenging and secondary predation (Deagle et al. 2019), we detected items such as *Solanum* spp., a resource for *M. persicae* in our laboratory rearings, reinforcing cautious interpretation of dietary breadth. Sample degradation may explain three individuals with no plant material and three with no taxa detected in August 2020. Distinguishing zoophagy from self‐detection remained challenging: ~80% of reads were lost after filtering, with most retained reads assigned to Miridae, complicating host identification and obscuring less frequent metazoans. Zoophagy is likely underestimated because metazoan DNA digests faster than *Streptophyta*, biasing animal‐to‐plant ratios; fecal samples often contain little or no DNA from prey consumed a few days prior (Deagle et al. 2005; Oehm et al. 2011), emphasizing the importance of prey‐consumption timing.

A consistent pattern across markers was the low number of order‐level assignments (Figure 1), reflecting limitations of rank‐specific classifiers like LCA, which assign ASVs only to the lowest rank confidently supported by divergence and reference coverage (Hleap et al. 2021). Short dietary fragments often allow broad placement or species‐level matches when diagnostic sites are present, but lack resolution for intermediate ranks, especially when reference databases are unevenly populated across taxa (Meglécz 2023). This combination explains the underrepresentation of order‐level identifications in both plant and animal datasets. Strawberry was detected in only one individual in July 2020, during peak plant host richness and peak strawberry harvest (Elmhirst 2005), and comprised 1.5% of ingested material. Although suboptimal, strawberry is ingested and used for oviposition and resting (Solà Cassi et al. 2024), and feeding trials showed 33.33% of both *L. lineolaris* and *N. americoferus* consumed it. Treatment thresholds range from 0.12 to 0.15 nymphs per flower stalk (Lambert et al. 2007) to 0.26 per blossom (Mailloux and Bostanian 1988; Ontario Ministry of Agriculture, Food and Rural Affairs [OMAFRA] 2009). Our data cannot infer infestation levels, and future work should assess whether strawberry detection correlates with field prevalence, including larval feeding contributions (Alberdi et al. 2019). Effective biological control may involve managing wild hosts or deploying summer trap crops such as buckwheat, canola, white mustard or safflower which bloom for 4–6 weeks (buckwheat; Kalinová et al. 2005) or ~6 weeks (canola; Lilley et al. 2019) (Dumont and Provost 2019; Wang et al. 2021). In 2020, 11.43% of individuals fed on at least one of these crops, peaking at 7.15% in July; that month, 30% consumed *Sinapis* spp., 40% *Brassica* spp., and 4.20% *Fagopyrum* spp. Non‐cultivated plants, *Ambrosia artemisiifolia* , *Amaranthus* spp. or *Verbascum thapsus* (L.) also function as trap crops (Dumont and Provost 2022). *Ambrosia* spp. ranked second in %FOO (peaking August–September 2019), *Amaranthus* spp. ranked fifth (peaking July–August), and 20% of individuals consumed *Verbascum* spp. in October. *Phellodendron* spp. and *Pteris* spp., the third and fourth most frequent taxa, peaked in autumn as *P. amurense* fruits matured (Ettinger et al. 2018). These species may serve as autumn trap crops when combined with vacuuming (Swezey et al. 2007; Dumont and Provost 2022). Additional IPM tools include selective herbicides or mowing (Snodgrass and Scott 2000; Snodgrass et al. 2006; Abel et al. 2007; Fleury et al. 2013; George et al. 2023), red sticky cards with pheromone blends (George et al. 2023), and introducing *N. americoferus* , which preyed on 100% of *L. lineolaris* in trials, though predator–prey interactions and nonconsumptive effects warrant caution.

Overall, metataxonomy clarified *L. lineolaris* feeding hosts and food web interactions in an omnivorous pest, providing evidence‐based guidance for sustainable, targeted IPM tailored to its diet and seasonal/sex‐specific dynamics.

## Author Contributions

**Mireia Solà Cassi:** conceptualization (lead), data curation (lead), formal analysis (lead), funding acquisition (equal), investigation (lead), methodology (lead), project administration (equal), resources (lead), software (lead), supervision (lead), validation (lead), visualization (lead), writing – original draft (lead), writing – review and editing (lead). **François Dumont:** funding acquisition (equal), project administration (equal), supervision (supporting), writing – original draft (supporting). **Eric Lucas:** funding acquisition (equal), project administration (equal), supervision (supporting), writing – original draft (supporting).

## Funding

This research was partially funded by the AgriScience program cluster from Agriculture and Agri‐Food Canada, and Mireia Solà Cassi received a MITACS Accélération postdoctoral scholarship (IT13625).

## Conflicts of Interest

The authors declare no conflicts of interest.

## Supporting information


**Data S1:** ece372954‐sup‐0001‐DataS1.docx.

## Data Availability

The data that support the findings of this study are openly available in Zenodo at: https://zenodo.org/badge/DOI/10.5281/zenodo.15650230.svg and https://doi.org/10.5281/zenodo.15650230.
