# Supplementary material for: Characterization of the Omnivorous Lygus lineolaris Diet in a Strawberry Field by Metataxonomy
Source: Ecol Evol. 2026 Jan 19;16(1):e72954. doi: 10.1002/ece3.72954 (PMC12813691; doi:10.1002/ece3.72954)
Supplement: Supplementary file 1 — Data S1: ece372954‐sup‐0001‐DataS1.docx. [file ECE3-16-e72954-s001.docx]

**Characterization of the omnivorous *Lygus lineolaris* diet in a strawberry field by metataxonomy**

Mireia Solà Cassi^1,2*^ (0000-0002-7701-6727), François Dumont^2^ (000-0001-7587-072X), Eric Lucas^1^ (0000-0003-4126-4988)

**Supplemental material**

Experimental Design

**Table S1.** Field samples. Collecting year, date, number of, *Lygus lineolaris* (TPB) and number of females (Num. F.) analyzed with metabarcoding.

| **Year** | **Date** | **Num. TPB** | **Num. F.** |
| --- | --- | --- | --- |
| 2019 | 30/07/19 | 10 | 8 |
|  | 23/08/19 | 10 | 5 |
|  | 17/09/19 | 10 | 6 |
|  | 18/10/19 | 10 | 5 |
| 2020 | 28/07/20 | 10 | 8 |
|  | 27/08/20 | 10 | 6 |
|  | 27/09/20 | 10 | 5 |

**Table S2.** Summary of the number of samples prepared for each validation category (Feeding Trials and Mock Samples). The table indicates the predator species used for DNA extraction and the diets offered during feeding trials; flower structures were provided for plant diets. Diet abbreviations are included to assist interpretation of validation plots. In the “Abbreviation name” column, labels ending in “_*” indicate that sample IDs include numerical suffixes (e.g., _1, _2, _3) to distinguish replicates.

| **Type of sample** | **Type of treatment** | **Species** | **Diet** | **Abbreviation name** | **Num. Samples** |
| --- | --- | --- | --- | --- | --- |
| Validation | Feeding trial | *N. americoferus* (Carayon)  (Hemiptera: Nabidae) | *Tetranychus urticae* (Koch) |  | 4 |
|  |  |  | *Lygus lineolaris* (Palisot de Beauvois) |  |  |
|  |  |  | *Myzus persicae* (Sulzer) | Mix N_* |  |
|  |  |  | *Fagopyrum esculetum* (Moench) |  |  |
|  |  |  | *Brassica napus* (L.) |  |  |
|  |  |  | *Fragaria x annanassa* (Duchesne) |  |  |
|  |  | *L. lineolaris*  (Palisot de Beauvois)  (Hemiptera: Miridae) | *Tetranychus urticae* |  | 4 |
|  |  |  | *Fagopyrum esculetum* |  |  |
|  |  |  | *Myzus persicae* | Mix L_* |  |
|  |  |  | *Fagopyrum esculetum* |  |  |
|  |  |  | *Brassica napus* |  |  |
|  |  |  | *Fragaria x annanassa* |  |  |
|  |  |  | *Fagopyrum esculetum* | F_* | 3 |
|  |  |  | *Myzus persicae* | M_* | 3 |
|  |  |  | Fastened TPB | Ø_* | 3 |
|  | Mock samples | Empty sample | - | Ø | 1 |
|  |  | *Cucumis sativus* (L.) | - | C | 1 |
|  |  | *Fagopyrum esculetum* | - | F | 1 |
|  |  | *Myzus persicae* | - | M | 1 |
|  |  | *Eupeodes americanus* (Wiedemann) | - | E | 1 |
|  |  | *Cucumis sativus* | - |  | 1 |
|  |  | *Fagopyrum esculetum* |  |  |  |
|  |  | *Myzus persicae (n=3)* |  | Mix Mock |  |
|  |  | *Eupeodes americanus* (6 legs) |  |  |  |

Primer’s selection

To identify a wide range of insects, spiders, and springtails DNA on the *L.lineolaris* diet, the cytochrome c oxidase subunit I (COI) region was targeted. First, with the most used primer set for arthropods detection: ZBJ-ArtF1c and ZBJ-ArtR2. Then, a complementary universal primer pair, that do not overlap the COI region and that is also widely used for arthropod detection was used: mlCOIintF and Fol-degen-rev (Leray et al. 2013, Masonick et al. 2019, Ducotterd et al. 2021) (Table S3).

To identify plant species (Streptophyta*)*, the universal P6 loop of the chloroplast trnL (UAA) intron region yielding an amplicon of 10-143bp was selected ^4^. This primer set, specified for angiosperms and gymnosperms detection, has produced very reliable PCR results ^5–7^. The second primer set selected for plants and fungus targeted the nuclear ITS2 region. This region has been suggested to be used as universal barcode region for plant and fungus as well as to be used for arthropods as complementary locus of COI ^8–10^ (Table 1).

| Custom database  **Table S3:** Number of records in each step of custom database construction and final number of groups obtained for plant trnL chloroplast and ITS2 nucleus regions, and animal mitochondrial COI Region. The process for database construction included: sequence downloading, duplicates removal, removal of missing taxonomic ranks and length filtering. Note that, for each target region, the length of ranks kept in the length filtering stage is described inside brackets. This length was selected according to the barcode literature on each target region. | | | | |
| --- | --- | --- | --- | --- |
|  | **Plant** | |  | **Animal** |
| **Process** | **trnL** | **ITS2** |  | **COI** |
| Downloaded sequences | 334,599 | 238,480 |  | 3,012,978 |
| Duplicates removal | 247,062 | 187,303 |  | 2,030,211 |
| Removal of missing Taxonomic Ranks | 245,987 | 186,910 |  | 1,402,179 |
| Length filtering (length of ranks kept) | 244,036 (<2,500) | 184,267 (>100-2000<) | | 1,396,567 (<120-2000>) |
| Total Number of Distinct Groups | 98,220 | 77,503 |  | 373,534 |

**Bioinformatic analyses**

Quality filtering

**Table S4.** Total Number of reads and percentage of reads retained in the quality filtering process using DADA2 for each primer set in the metabarcoding analysis (Target regions in parentheses).

|  |  | **Plant** | |  | **Animal** | |
| --- | --- | --- | --- | --- | --- | --- |
| **Process** |  | g/h (trnL) | ITSu3/ITSu4 (ITS2) |  | ZBJ‐ArtF1c/ZBJ‐ArtF2c (COI) | mICOIintF/Fol-degen-rev (COI) |
| **Input reads** | | 962,077 | 1,108,964 |  | 2,284,243 | 380,185 |
| **Filtered reads** | | 720,437 | 542,522 |  | 1,994,563 | 338,268 |
| **Denoised reads Forward** | | 718,798 | 540,668 |  | 1,994,180 | 337,638 |
| **Denoised reads Reverse** | | 720,099 | 541,019 |  | 1,994,150 | 337,494 |
| **Merged amplicons** | | 689,543 | 338,939 |  | 1,987,255 | 331,982 |
| **Non-chimeric reads** | | 674,813 | 264,424 |  | 1,965,009 | 330,534 |
| **% reads retained** | | 73.2 | 22.6 |  | 86.9 | 89.3 |

For Streptophyta, after quality filtering, the primer set g/h experienced a 26.8% of reads lost, resulting in a final count of 674,813 reads and a mean coverage of 6,546 ± 344.46 reads per sample. In contrast, the primer set ITSu3/ITSu4 had a notably higher percentage of lost reads (51.1%), with 264,424 reads retained and a mean coverage of 2,655 ± 102.53 reads per sample (Table S4).

In addition, after filtering reads with the highest read abundance in negative controls, 1% of read depth was lost with ITS2 while 10.77% with trnL.

For Metazoan, after quality filtering, the primer set ZBJ-ArtF1c/ZBJ-ArtF2c, displayed a 13.1% of reads lost, retained 1,965,009 reads and a mean coverage of 17,461 ± 1,649.83 reads per sample while mICOIintF/Fol-degen-rev presented 10.7% of read lost retained 330,534 reads and a mean coverage of 2,229 ± 362.48 reads per sample.

After excluding self-host DNA from *L. lineolaris* and *N. americoferus* samples, 94.92% and 81.65% of read depths were lost for ZBJ‐ArtF1c/ZBJ‐ArtF2c and mICOIintF/Fol-degen-rev primer sets, respectively.

Primers resolution

**Table S5.** Primer agreement at five taxonomic ranks Plant and Animal assignments. Shown are the number of items assigned by each primer, their overlap, and the number of concordant and conflicting assignments within the overlap. Full lists of conflicting items are provided in Table S6.

|  |  | **Plant** | | | | |  | **Animal** | | | | |
| --- | --- | --- | --- | --- | --- | --- | --- | --- | --- | --- | --- | --- |
| Rank |  | ITS2 | trnL | Overlap | Concordant | Conflicting |  | zeale | mcoi | Overlap | Concordant | Conflicting |
| Species |  | 32 | 3 | 1 | 0 | 1 |  | 11 | 2 | 0 | 0 | 0 |
| Genus |  | 50 | 8 | 3 | 0 | 3 |  | 16 | 2 | 0 | 0 | 0 |
| Family |  | 50 | 17 | 9 | 0 | 9 |  | 18 | 2 | 0 | 0 | 0 |
| Order |  | 50 | 17 | 9 | 0 | 9 |  | 18 | 2 | 0 | 0 | 0 |
| Class |  | 61 | 17 | 10 | 8 | 2 |  | 18 | 2 | 0 | 0 | 0 |

**Table S6.** Conflicting Streptophyta assignments between ITS2 and trnL across taxonomic ranks. No conflicts were detected for Metazoa (see Table S5).

| **TPB_id** | **Rank** | **ITS2** | **trnL** |
| --- | --- | --- | --- |
| TP89 | Species | *Dioscorea_japonica;Phellodendron_amurense* | *Hordeum_murinum* |
| TP140 | Genus | *Phellodendron* | *Chenopodium* |
| TP47 |  | *Amaranthus* | *Solanum* |
| TP89 |  | *Dioscorea;Phellodendron* | *Hordeum;Plantago;Solanum* |
| TP117 | Family | *Rutaceae* | *Asteraceae* |
| TP138 |  | *Brassicaceae* | *Rosaceae* |
| TP140 |  | *Rutaceae* | *Chenopodiaceae* |
| TP142 |  | *Pteridaceae* | *Brassicaceae* |
| TP144 |  |  |  |
| TP17 |  | *Amaranthaceae* |  |
| TP20 |  | *Asteraceae* |  |
| TP47 |  | *Amaranthaceae* | *Solanaceae* |
| TP89 |  | *Dioscoreaceae;Rutaceae* | *Plantaginaceae;Poaceae;Solanaceae* |
| TP117 | Order | *Sapindales* | *Asterales* |
| TP138 |  | *Brassicales* | *Rosales* |
| TP140 |  | *Sapindales* | *Caryophyllales* |
| TP142 |  | *Polypodiales* | *Brassicales* |
| TP144 |  |  |  |
| TP17 |  | *Caryophyllales* |  |
| TP20 |  | *Asterales* |  |
| TP47 |  | *Caryophyllales* | *Solanales* |
| TP89 |  | *Dioscoreales;Sapindales* | *Lamiales;Poales;Solanales* |
| TP142 | Class | *Polypodiopsida* | *Magnoliopsida* |
| TP144 |  |  |  |

*Streptophyta*

Both plant markers exhibited varying levels of taxonomic resolution which was successfully complemented when used together with the plant multimarker (Fig. 1). The ITSu3/ITSu4 marker (ITS2 region) detected a total of 394 occurrences, of which 32.99% remained unclassified or were classified at the phylum Streptophyta level, and 22.08% of the samples couldn't be classified beyond Cl. Magnoliopsida. Conversely, the g/h marker (trnL region) identified 204 occurrences, with 17.25% unable to be classified beyond Class Magnoliopsida. In the case of the ITSu3/ITSu4 primer, the analysis of gut content from field-collected *L. lineolaris* yielded 85 plant occurrences beyond Class level, spanning 21 different plant taxa across 2 classes, 9 orders, 11 families, 16 genera, and 12 species. Furthermore, 52.17% of the taxa were identified at the species level, 39.13% at the genus level and 0% at family or order level and 4.35% could not be identified beyond Class level (Fig. 1). In contrast, the trnL region targeted by the g/h primer set revealed 141 plant occurrences in the gut content of *L. lineolaris* collected from the field and identified beyond class taxonomic level. These occurrences represented 21 different plant taxa, distributed across 2 classes, 9 orders, 12 families, 14 genera, and 5 species. Notably, 22.73% of the taxa were identified at the species level, 45.45% at the genus level, 27.70% at the family level, 0% at order level and 4.55% could not be identified beyond Class Magnoliopsida. The use of plant multimaker converged in 216 occurrences beyond Class taxonomic level spanning 40 unique plant taxa. The analysis of the gut content of *L. lineolaris* collected in the field revealed 40.00% and 42.50% of identifications at the species and genus level, respectively while 15.00% at family level and 2.50% at class level (Fig. 1).

*Metazoa*

The percentage of unknown occurrences was 57.45% for ZBJ‐ArtF1c/ZBJ‐ArtF2c while 81.91% for mICOIintF/Fol-degen-rev. In addition, the primer set mICOIintF/Fol-degen-rev, was unable to identify taxa beyond Insects Class in 85.71% of the occurrences of the field collected samples. The ZBJ‐ArtF1c/ZBJ‐ArtF2c primer set exhibited enhanced taxonomic resolution compared to mICOIintF/Fol-degen-rev. Notably, 41.67% of the taxa were identified at the species level, 50.00% at the genus level and 8.33% at the family level. For the primer set mICOIintF/Fol-degen-rev, 33.33% of occurrences were identified at Species level, another 33% at genus while the rest remained at class level (Fig. 1). Specifically, the analysis of *L. lineolaris* gut content with the ZBJ‐ArtF1c/ZBJ‐ArtF2c primer set revealed 36 arthropod occurrences beyond Class taxonomic level. These belonged to 12 distinct taxonomic units, distributed across 2 classes, 5 orders, 11 families, 10 genus and 6 species. On the other side, mICOIintF/Fol-degen-rev primer set identified 14 occurrences in the gut content of field collected *L. lineolaris.* Only 2 of them were classified beyond the Insects Class level. They belonged to two different orders; one was identified at species level while the other at genus level (Fig. 1). Interestingly, the taxa *F. occidentalis* which was part of the control treatment, and *Nabis sp*. found in the field collected *L.lineolaris*, were uniquely detected by the mICOIintF/Fol-degen-rev primer set (Table 4). The use of animal multimaker in the field collected samples, converged in 27 occurrences beyond Class taxonomic level spanning 13 unique Arthropoda taxa were 35.71% of identifications were resolved at the species level, 50.00% at the genus level and 7.14% at both family and class level. (Fig. 1)

Validation samples results

*Streptophyta*

*Fagopyrum sp.* was effectively detected in the mock samples when presented alone or in combination with *Cucumis sp.,* *Eupeodes spp.,* and *Myzus spp*. However, *Cucumis sp*. was not detected when analyzed either single or combined. Furthermore, *Solanum sp.*, part of our *M. persicae* lab breeding diet, was detected in the aphids' gut content. In choice feeding trials, *Fagoyrum spp.* was identified in 66.66% of both *L. lineolaris* and *N. americoferus* individuals. *Brassica spp.* was found in 100% of *N. americoferus* individuals and in 66% of tested *L. lineolaris* individuals. *Fragaria spp.* was discovered in 33.33% of the individuals, whether in Lygus or Nabis gut content. When single diets were offered to *L. lineolaris*, cross-contamination with *Brassica spp.* was observed in 33.33% of *L. lineolaris* individuals that were either fastened or fed with *Fagopyrum spp.* Moreover, *Ambrosia spp.* was detected when aphids were offered to *L. lineolaris*, revealing a second source of cross-contamination (Table S2, Fig. S1).

*Metazoa*

All metazoans within the mock samples were successfully identified when examined individually. However, when *Eupeodes spp*. and *Myzus spp.* were combined with *Cucumis spp.* and *Fagopyrum spp.*, only *Eupeodes spp.* was detected among these taxa. Additionally, *Franklinella spp.* was also identified in this combination, revealing common infestations within our greenhouses. Other typical infestations in our greenhouses, namely *Bradisia spp.* and *Aphidius spp*., were identified in feeding trials with *N. americoferus* and *L. lineolaris*. Additionally, *Bembidion spp.*, commonly found in strawberry fields, was unexpectedly detected in the gut content of a *L. lineolaris* of a feeding trial where aphids, spider mites, buckwheat, canola, and strawberry were offered. *L. lineolaris* exhibited 100% presence in the guts of *N. americoferus* (Table S2, Fig. S1).


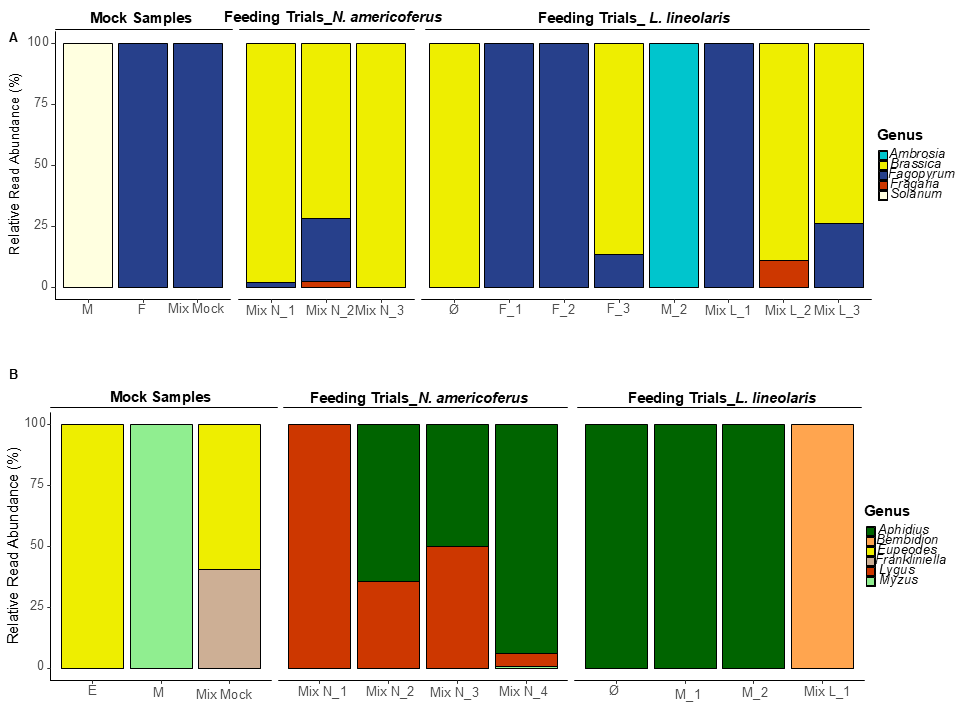


**Figure S1.** Relative read abundance (%RRA) of validation samples at the genus level for (A) Streptophyta and (B) Metazoa. Samples are grouped according to validation type: mock samples, feeding trials with *Nabis americoferus*, and feeding trials with *Lygus lineolaris*. Sample names correspond to the organism from which DNA was extracted and follow the naming and abbreviation scheme detailed in Table S2. Replicate samples are indicated by numerical suffixes (e.g., _1, _2, _3).

Supplemental information about contamination

The following missclassified taxa were removed from analyses. These instances included *Mojiangia oreophila* (Asterale: Asteraceae) with a frequency of occurrence (FFO) of 23.4%, *Theobroma spp*. and *T. microcarpum* (Malvale: Malvaceae) with an overall FOO of 1.6%, and *Brassica gravinae,* which was reclassified as *Brassica spp*. For metazoans, only taxa identified above Genus level were retained. Notably, *Artemia spp*. (Anostraca: Artemiidae) at 27.3% and *Plodia spp.* (Lepidoptera: Pyralidae) at 5,5% FOO were excluded from the analyses due to potential misclassification.

**References**

(1) Leray, M.; Yang, J. Y.; Meyer, C. P.; Mills, S. C.; Agudelo, N.; Ranwez, V.; Boehm, J. T.; Machida, R. J. A New Versatile Primer Set Targeting a Short Fragment of the Mitochondrial COI Region for Metabarcoding Metazoan Diversity: Application for Characterizing Coral Reef Fish Gut Contents. *Frontiers in zoology* **2013**, *10* (1), 1–14.

(2) Masonick, P.; Hernandez, M.; Weirauch, C. No Guts, No Glory: Gut Content Metabarcoding Unveils the Diet of a Flower‐associated Coastal Sage Scrub Predator. *Ecosphere* **2019**, *10* (5), e02712.

(3) Ducotterd, C.; Crovadore, J.; Lefort, F.; Rubin, J.; Ursenbacher, S. A Powerful Long Metabarcoding Method for the Determination of Complex Diets from Faecal Analysis of the European Pond Turtle (Emys Orbicularis, L. 1758). *Molecular ecology resources* **2021**, *21* (2), 433–447.

(4) Taberlet, P.; Coissac, E.; Pompanon, F.; Gielly, L.; Miquel, C.; Valentini, A.; Vermat, T.; Corthier, G.; Brochmann, C.; Willerslev, E. Power and Limitations of the Chloroplast Trn L (UAA) Intron for Plant DNA Barcoding. *Nucleic acids research* **2007**, *35* (3), e14–e14.

(5) Valentini, A.; Pompanon, F.; Taberlet, P. DNA Barcoding for Ecologists. *Trends in Ecology & Evolution* **2009**, *24* (2), 110–117. https://doi.org/10.1016/j.tree.2008.09.011.

(6) Jurado-Rivera, J. A.; Vogler, A. P.; Reid, C. A. M.; Petitpierre, E.; Gómez-Zurita, J. DNA Barcoding Insect–Host Plant Associations. *Proceedings of the Royal Society B: Biological Sciences* **2009**, *276* (1657), 639–648. https://doi.org/10.1098/rspb.2008.1264.

(7) Mogren, C. L.; Benítez, M.-S.; McCarter, K.; Boyer, F.; Lundgren, J. G. Diverging Landscape Impacts on Macronutrient Status despite Overlapping Diets in Managed (Apis Mellifera) and Native (Melissodes Desponsa) Bees. *Conservation physiology* **2020**, *8* (1), coaa109.

(8) Yao, H.; Song, J.; Liu, C.; Luo, K.; Han, J.; Li, Y.; Pang, X.; Xu, H.; Zhu, Y.; Xiao, P.; Chen, S. Use of ITS2 Region as the Universal DNA Barcode for Plants and Animals. *PLoS One* **2010**, *5* (10), e13102. https://doi.org/10.1371/journal.pone.0013102.

(9) Chen, D.; Xue, Y.; Chen, S.; Fink, D.; Gomes, C. Deep Multi-Species Embedding. *arXiv preprint arXiv:1609.09353* **2016**.

(10) Sponsler, D. B.; Grozinger, C. M.; Richardson, R. T.; Nurse, A.; Brough, D.; Patch, H. M.; Stoner, K. A. A Screening-Level Assessment of the Pollinator-Attractiveness of Ornamental Nursery Stock Using a Honey Bee Foraging Assay. *Scientific reports* **2020**, *10* (1), 1–9.
